# Ameliorating Effect of Transcutaneous Electroacupuncture on Impaired Gastric Accommodation in Patients with Postprandial Distress Syndrome-Predominant Functional Dyspepsia: A Pilot Study

**DOI:** 10.1155/2015/168252

**Published:** 2015-05-03

**Authors:** Feng Xu, Yan Tan, Zhihui Huang, Nina Zhang, Yuemei Xu, Jieyun Yin

**Affiliations:** ^1^Division of Gastroenterology, Yinzhou Hospital Affiliated to Medical School of Ningbo University, Ningbo 315000, China; ^2^Division of Gastroenterology, Affiliated Hospital of Hainan Medical College, Haikou 571000, China; ^3^Ningbo Pace Translational Medical Research Center, Beilun, Ningbo 315000, China; ^4^Department of Gastroenterology, Sir Run Run Shaw Hospital, School of Medicine, Zhejiang University, Hangzhou 310000, China

## Abstract

Patients with functional dyspepsia (FD) have both reduced gastric accommodation and impaired gastric motility that are difficult to treat. The aim of this study was to investigate the therapeutic potential of transcutaneous electroacupuncture (TEA) for both of these disorders in FD patients. Acute experiments were performed in FD patients to study the effect of TEA and sham-TEA on gastric accommodation assessed by a nutrient drink test and gastric motility assessed by the measurement of the electrogastrogram (EGG). TEA or sham-TEA was performed via cutaneous electrodes at acupoints ST36 and PC6 or sham-points nonacupoints. It was found that (1) gastric accommodation (maximum tolerable volume) was reduced in FD patients compared with the controls (*P* < 0.03). TEA improved gastric accommodation in FD patients (*P* < 0.02). (2) Acute TEA significantly increased the percentage and power of normal gastric slow waves in the fed state assessed in the FD patients by the EGG in comparison with sham-TEA. (3) TEA increased vagal activity assessed by the spectral analysis of the heart rate variability in the fed state in FD patients. It was concluded that needleless method of transcutaneous electroacupuncture may have a therapeutic potential for treating both impaired gastric accommodation and impaired gastric motility in patients with FD.

## 1. Introduction

The prevalence of functional dyspepsia (FD) is high but the treatment options have been limited [1]. Patients with FD complain about symptoms of epigastric pain, abdominal fullness, early satiety, and abdominal discomfort. Pathophysiologies of FD include visceral hypersensitivity, reduced gastric accommodation, and impaired gastric motility, such as gastric dysrhythmia, antral hypomotility, and delayed gastric emptying [2].

Gastric accommodation is mediated by the vagal nerve. Upon food ingestion, the vagal nerve is activated and nitric oxide is released, resulting in a relaxation of the stomach. This relaxation reflex accommodates ingested food without causing an increase in gastric pressure [3]. Impaired gastric accommodation leads to early satiety and postprandial fullness, possibly attributed to weakening of the vagal nerve.

After the patients with GI disorder eat food, a series of indigestion symptoms of early satiety and abdominal distension will appear due to insufficient relaxation of proximal gastric and intragastric pressure increasing. About 40% to 70% of FD patients have proximal GI disorder [4]. Accordingly, treatment for impaired gastric accommodation is of great clinical significance [5, 6].

Common treatment options for FD include dietary measures, pharmacologic treatments, such as acid-suppression drugs, prokinetic agents, fundus relaxing drugs, and antinociceptive agents, and psychological interventions [7–16]. In general, targeted therapies directed at the underlying pathophysiology are desirable. However, efficacy of the therapy is usually very limited due to multiple symptoms and pathophysiologies in individual patients. For example, a patient may have impaired accommodation and delayed gastric empting at the same time; in this case, prokinetic agents can be used to treat delayed gastric emptying but would worsen the symptoms related to gastric accommodation because available prokinetics often impair gastric accommodation. For the same reason, fundus relaxing drugs may be used for treating impaired accommodation; however, these drugs may delay gastric emptying because they relax muscles. The treatment approach to the patients with hypersensitivity to gastric distension has not been established. Antidepressants are commonly used in functional gastrointestinal disorders and were thought to exert a visceral analgesic rather than an antidepressant effect. However, studies of the effects of antidepressants on visceral sensitivity are rare, and the existing data on visceral sensitivity are controversial [14, 15].

Acupuncture has been used to treat gastrointestinal symptoms in China for thousands of years. The most commonly used acupuncture points (acupoints) for the treatment of gastrointestinal symptoms are Neiguan (PC6) and Zusanli (ST36). In clinical research, manual acupuncture is commonly replaced with electroacupuncture that is more reproducible. In a comparative study, electroacupuncture was found to be as effective as manual acupuncture in treating pain [17]. Electroacupuncture at ST36 and PC6 has been documented to increase the regularity of gastric slow waves and accelerate gastric emptying of liquids in animals [18]. In recent studies, electroacupuncture was reported to accelerate gastric emptying of solids and improve dyspeptic symptoms and gastric dysrhythmia in patients with FD and patients with diabetes [19, 20] and similar beneficial effects can be observed in patients with FD when electroacupuncture is applied without needles or a method called transcutaneous electroacupuncture (TEA) [21]. TEA is a completely noninvasive method which is readily accepted by patients. However, it is unknown whether TEA is able to treat both reduced gastric accommodation and impaired gastric motility in patients with FD.

The aims of this study were to investigate the therapeutic potential of TEA for patients with FD by assessing its acute effects on gastric accommodation assessed by a noninvasive nutrient drink test and gastric motility assessed by noninvasive electrogastrography as well as dyspeptic symptoms and to explore vagal mechanisms involved with TEA.

## 2. Materials and Methods

### 2.1. Subjects

Eight FD patients with postprandial distress syndrome and 8 healthy volunteers aged 21 to 65 years old were recruited in this study. Patients included fulfilled Rome III criteria for FD postprandial distress syndrome [1]. Patients who were unable to give informed consent; were taking prokinetic, anticholinergic, or dopaminergic agents during the experimental period; had a history of gastrointestinal surgery; were pregnant or preparing to conceive a child; had diabetes; and were allergic to skin preparation and familiar with acupoints and their functions were excluded from the study. Inclusion criteria of healthy volunteers include no history of supreme gastrointestinal diseases, including peptic ulcer disease, gastroesophageal reflux disease, and hepatobiliary and pancreatic diseases, a history of abdominal surgery, no history of alcohol abuse, no serious systemic illness and possible malignancy, and usually no dyspeptic symptoms, including upper abdominal pain, upper abdominal discomfort, postprandial fullness, upper abdominal swelling, early satiety, nausea, vomiting, excessive belching, and heartburn. All general information including height, weight, address, and relating medical history is recorded and all the subjects had signed the informed consent prior to the study. The experimental protocol was approved by the ethical committee of Yinzhou People's Hospital and all the subjects signed the consent form before participation.

### 2.2. Experimental Protocol

All subjects were studied in the morning after a 12-hour fast. Each subject was studied for two sessions in a randomized order: TEA and sham-TEA sessions. The experiment protocol was as follows: 30-minute baseline recording, 30-minute TEA/sham-TEA treatment in the fasting state, and then a satiety drinking test conducted with a liquid meal of  Ensure (0.95 kcal/mL) with TEA/Sham-TEA. After the completion of satiety drinking test, there was a 30-minute recovery period with TEA/sham-TEA. Electrogastrogram (EGG) and electrocardiogram (ECG) were recorded during the entire experimental period except during the satiety drinking test.

### 2.3. Transcutaneous Electroacupuncture

Acupoints ST36 (Zusanli) and PC6 (Neiguan) were used in the TEA session. ST36 is located at the place of 4-finger-breadth measuring down from the outer eye of the knee between the fibula and the tibia, 1-finger-breadth measurement beside the tibia; PC6 is located at the place of one-sixth of remote end and five-sixths of proximal end of the connection stripe between the transverse wrist crease and cubical crease. The stimulation was delivered by two portable neuromodulation devices at ST36 and PC6, respectively (SNM-FDC01, Ningbo Maida Medical Device Inc.). The stimulation parameters were chosen as 2s-on, 3s-off, 25 Hz, 0.6 ms, and amplitude of 2 mA to 10 mA depending on tolerance of the subject, which was shown to improve gastrointestinal symptoms in patients with diabetic gastroparesis [22]. In the sham-TEA group, the sham-acupoint for PC6 was located at about 15–20 cm away from PC6 (up to the elbow and outside coastal margin of the forearm not on any meridian) and the sham-point for ST36 was located at 10–15 cm down from and to the lateral side of ST36 not on any meridian. The stimulation parameters used for sham-TEA were the same as in the TEA.

### 2.4. Satiety Drinking Test

The gold-standard method of assessing gastric accommodation is the barostat method. However, this method is not well tolerated by patients due to intubation of a plastic bag into the stomach. Recently, the satiety drinking test has been used as a surrogate for the measurement of gastric accommodation [23]. A higher volume taken by the subject is indicative of a higher gastric accommodation. In this method, after an overnight fast, the subject was instructed to take Ensure (0.95 kcal/mL) at a rate of 120 mL every 4 minutes (average 30 mL/min) until the subject reported to reach satiety (complete fullness). During the test, each subject was asked to score satiety at a 5-minute interval using following scores: 0: no symptoms; 1: initial satiety (threshold); 2: mild; 3: moderate; 4: severe; 5: maximum or intolerable satiety. When reaching score 5, the subject was asked to stop drinking and the total volume drunk was recorded, which reflected the maximum tolerable volume (MTV).

### 2.5. Assessment of Autonomic Function

The electrocardiogram (ECG) was recorded using a one-channel amplifier with a cut-off frequency of 100 Hz (Ningbo Maida Medical Device Inc., Ningbo, China) from two active ECG electrodes and one ground electrode. The two leads were attached to the right edge of the sternum and apex of the subjects and the ground to the right side of the abdomen. The heart rate variability (HRV) signal was derived from the ECG recording using a special program developed [24] by identifying R peaks, calculating and interpolating the R-R intervals so that the time interval between consecutive samples was equal and finally downsampling the interpolated data to a frequency of 1 Hz.

Overall power spectral analysis was applied to the HRV signal and the power in each frequency subband was calculated. The power in the low frequency band (0.04–0.15 Hz), LF, represents mainly sympathetic activity and part of parasympathetic activity. The power in the high frequency band (0.15–0.50 Hz), HF, stands purely for parasympathetic or vagal activity. For LF and HF, standard calculations were done, respectively, by LF/(HF + LF) and HF/(HF + LF) [25].

### 2.6. Recording and Analysis of Electrogastrogram (EGG)

The gastric myoelectrical activity was recorded using a 4-channel electrogastrogram (EGG) device (MEGG-04A, Ningbo Maida Medical Device Inc., Ningbo, Zhejiang, China) via 6 cutaneous electrodes described as follows. First, the abdomen where electrodes were to be placed was cleaned with a special gel (Nuprep, Weaver and Company, Aurora, USA); then conductive gel (Ten20, Weaver and Company, Aurora, USA) was applied to the cleaned skin area to reduce skin-electrode impedance. After this, six cutaneous electrodes were placed on the abdominal skin surface based on a previously established method [2]. The subject was in a supine position for the EGG recordings and talking, reading, or sleeping was not allowed.

Established EGG parameters were derived from the EGG signals using a spectral analysis software package (Ningbo Maida Medical Device Inc., Ningbo, China) after a careful deletion of motion artifacts [26, 27]: (1) dominant frequent and power, representing the frequency and amplitude of gastric slow waves; (2) percentage of normal 2–4 cycles/min slow waves, representing the regularity of gastric slow waves; (3) postpreprandial ratio of EGG dominant power, standing for postprandial increase in gastric motility.

### 2.7. Assessment of Dyspeptic Symptoms

Gastric cardinal symptom index was used to assess dyspeptic symptoms at baseline and after the acute TEA or sham-TEA [28]. These included upper abdominal pain, upper abdominal discomfort, postprandial fullness, upper abdominal swelling, early satiety, nausea, vomiting, excessive belching, and heartburn. Each symptom was graded based on severity: grade 0: no symptoms; grade 1: mild; grade 2: moderate; grade 3: severe.

### 2.8. Statistical Analysis

Results are expressed as mean ± standard deviation. Paired Student's* t*-test was used to study the difference between TEA and sham-TEA and between baseline and after the acute treatment using SPSS 16.0 statistical software. *P* < 0.05 was considered statistically significant.

## 3. Results

### 3.1. Effects of TEA on Gastric Accommodation

**Figure 1:**
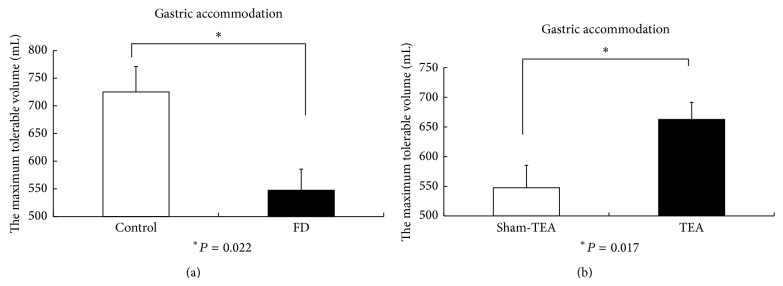
(a) The maximum tolerable volume (gastric accommodation) in normal controls and patients with FD. (b) The maximum tolerable volume after TEA and sham-TEA.

FD patients showed a reduced gastric accommodation that was improved with acute TEA. The MTV was 725 ± 46 mL in the normal control group and 548 ± 38 mL in the FD patients (*P* = 0.022; see Figure 1(a)). Acute TEA increased the MTV in the FD patients to 663 ± 29 mL (*P* = 0.007, versus baseline), whereas the sham-TEA did not increase the MTV in patients with FD (549 ± 36 mL after sham-TEA (*P* = 0.121 versus 700 mL)). There was a difference (*P* = 0.017) in MTV in the FD patients after TEA and sham-TEA (Figure 1(b)).

### 3.2. Effects of TEA on Gastric Slow Waves

**Figure 2:**
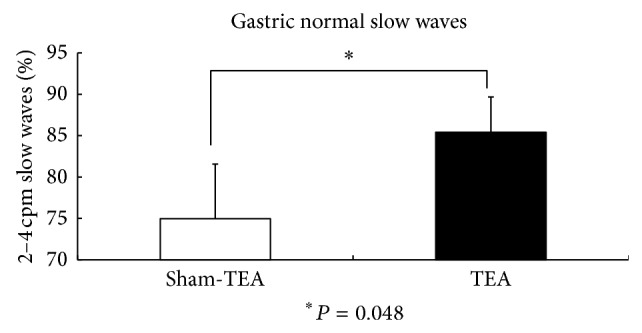
TEA improved the percentage of normal 2–4 cycles/min slow waves in the fed state.

**Figure 3:**
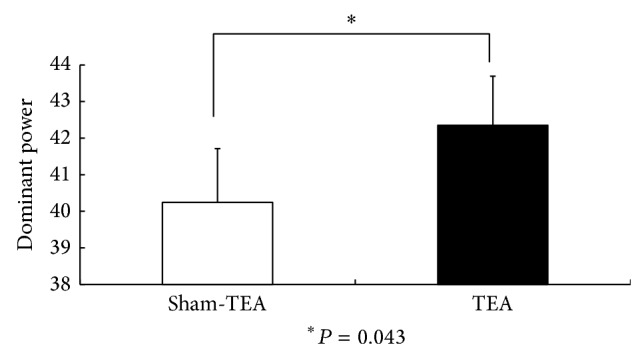
The comparison of EGG dominant power in the fed state after sham-TEA and TEA.

**Figure 4:**
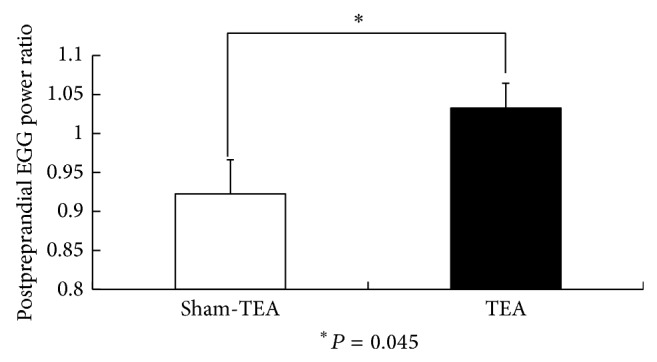
The comparison of postpreprandial EGG power ratio between sham-TEA and TEA.

The EGG recording was found to be normal in 2 patients but abnormal in 6 patients with FD (percentage of normal slow waves below 65% in either fasting or fed state or this was a postprandial decrease in dominant power). The major EGG parameters in the TEA and sham-TEA sessions are shown in Table 1. TEA improved the percentage of normal 2–4 cycles/min slow waves in the fed state (Figure 2) and also increased the dominant EGG power in the fed state (Figure 3). The postpreprandial EGG power ratio was significantly higher in the TEA sessions than in the sham-TEA session (Figure 4).

### 3.3. TEA Enhanced Vagal Activity

**Figure 5:**
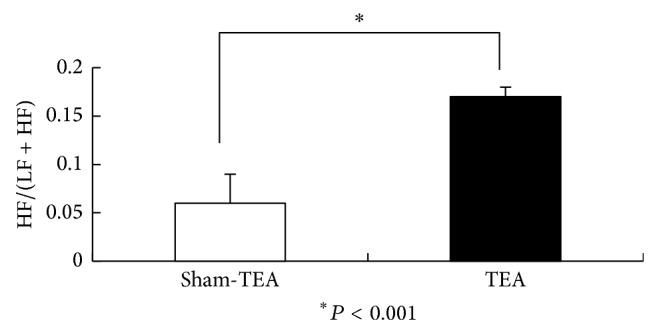
The vagal activity HF/(LF + HF) assessed by the spectral analysis of HRV in patients with FD treated with sham-TEA and TEA.

The acute TEA significantly increased the vagal activity in the 30 min postprandial period in patients with FD assessed by the spectral analysis of HRV. The HF/(LF + HF) was 0.17 ± 0.01 in the TEA session and 0.06 ± 0.03 in the sham-TEA session (*P* < 0.001) (see Figure 5).

### 3.4. Effects of Acute TEA on Dyspeptic Symptoms

**Figure 6:**
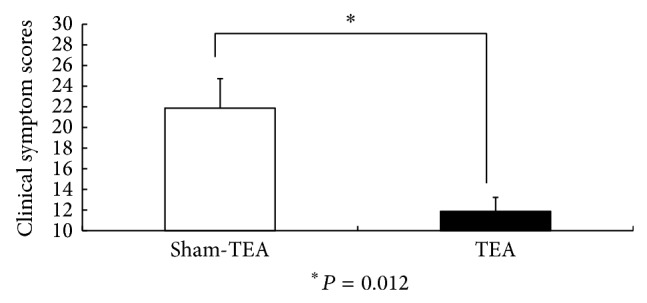
The clinical symptom scores in FD patients after TEA and sham-TEA treatment.

Acute TEA improved the dyspeptic symptoms in the FD patients. The mean total symptom score was 23.5 ± 2.9 at baseline and decreased significantly to 11.9 ± 1.4 (*P* = 0.007 versus baseline) after TEA but was 21.9 ± 2.9 after sham-TEA (*P* = 0.102 versus baseline). There was a significant difference in the clinical symptom scores between the FD patients after true treatment and those after sham treatment (Figure 6).

## 4. Discussion

In this study, we found that acute TEA at the acupoints of ST36 and PC6 improved gastric accommodation and enhanced postprandial gastric slow waves in patients with FD (increased the amplitude and regularity of slow waves). A concurrent increase in vagal activity was also noted with the acute TEA, suggesting a vagal mechanism. Acupuncture or electroacupuncture has been used to treat the symptoms of upper abdomen, such as nausea and vomiting. Hu et al. [29] reported that electroacustimulation at point PC6 reduced significantly the severity of the symptoms of motion sickness. The number of emetic episodes induced by morphine [30] or cyclophosphamide [31] was significantly reduced by electroacupuncture at the PC6 point in ferrets. Electroacupuncture at both the PC6 and the ST36 points reduced the incidence of vomiting induced by vasopressin in dogs [32]. A few papers reported the effect of acupuncture or electroacupuncture on dyspeptic symptoms in patients with FD. In one study with FD patients, acupuncture was demonstrated to be effective in reducing dyspeptic symptoms [19].

While electroacupuncture has been proven effective in treating certain functional gastrointestinal diseases, the insertion of acupuncture needles is required and the treatment has to be done at a doctor's office. The method proposed in this study, TEA, did not require the insertion of any needles and the procedure could be done by the patient at his/her home. This was more attractive than electroacupuncture and was well accepted by the patients as the compliance of the therapy was 100%; none of the patients quitted the study. It is similar to transcutaneous electrical nerve stimulation except that the stimulation electrodes in this study were placed on the acupuncture points related to the targeting disorder. Liu et al. [33] found that a two-week treatment of TEA at ST36 and PC6 significantly improved dyspeptic symptoms and increased vagal activity in patients with FD. These findings were in agreement with the present study. However, the effect of TEA on gastric accommodation was not previously investigated.

Impaired gastric accommodation in FD is difficult to treat because it requires the use of muscle relaxant. The use of muscle relaxant, however, worsens impaired gastric motility that is common in FD. In this study, acute TEA significantly and substantially improved gastric accommodation while concurrently improving gastric motility assessed by electrogastrography. This is an attractive strength of the proposed method of TEA. As stated earlier, impaired gastric accommodation is associated with symptoms of early satiety and postprandial fullness and bloating. The TEA-induced increase in gastric accommodation could lead to improvement in these symptoms. Although exact mechanisms involved in the increase of gastric accommodation were unknown, the concurrent increase in vagal activity noted in this study was believed to play a major role.

Electrogastrography has previously been shown to be an accurate and reliable method for studying gastric myoelectrical activity. Several studies have reported EGG abnormalities in FD patients [34, 35]. Meanwhile, it is known that electroacupuncture may affect gastric myoelectrical activity. A number of studies have investigated the effect of electroacupuncture on the gastric slow waves. Ouyang et al. [18] showed that electroacupuncture at ST36 and PC6 increased the regularity of gastric slow waves in both the proximal and distal stomach. Chang et al. [20] found that electrical stimulation at ST36 increased the percentage of normal EGG frequency and decreased the percentage of tachygastrial frequency in diabetic patients. Electroacupuncture at ST36 and PC6 increased the percentage of regular slow waves, resulting in the normalization of dysrhythmia in healthy human [36]. However, Liu et al. [33] study showed that TEA at ST36 and PC6 points did not change the EGG parameters in the patients with FD, suggesting that TEA may not treat disorders induced by gastric myoelectrical disturbances. In this study, however, we found that acute TEA at the acupoints of ST36 and PC6 improved gastric slow waves in the postprandial state. It should be noted that in this study the EGG in the postprandial state was recorded after the maximum ingestion of a nutrient liquid meal. This was apparently different from the postprandial recording after a regular meal.

Altered HF and LF/HF in the spectral analysis of HRV in patients with FD have been previously reported [37, 38]. It has been proposed that the autonomic dysfunctions could play a role in the development of disturbed gastric motility and perception. Spectral analysis of the HRV is a noninvasive and simple method for the quantitative evaluation of autonomic activity [39, 40]. We used this method to evaluate the effect of acute TEA on HRV in patients with FD and found a significant increase in HF after the TEA treatment. This result is in good agreement with others reported previously [18, 33, 41]. Although we did not have proof that this was responsible for the improvement in dyspeptic symptoms, it was consistent with the hypothesis that the visceral effects of TEA are at least partially mediated by the autonomic nerve pathway.

In summary, acute TEA at ST36 and PC6 significantly improves gastric accommodation and postprandial slow waves as well as dyspeptic symptoms, possibly mediated via the vagal mechanisms. Chronic clinical studies are warranted to establish clinical role of this noninvasive method of TEA for treating FD.

## Figures and Tables

**Table 1:** Effects of acute TEA treatment on EGG in patients with functional dyspepsia in the study.

	Session	
	TEA	Sham-TEA
Dominant frequency (cpm)		
Fasting	3.02 ± 0.03	3.04 ± 0.06
Postprandial	2.84 ± 0.07	3.25 ± 0.10
Dominant power (dB)		
Fasting	33.98 ± 1.58	34.46 ± 1.75
Postprandial	42.35 ± 1.35	40.24 ± 1.47^*^
Percentage of normal slow waves (%)		
Fasting	82.6 ± 3.1	83.7 ± 2.7
Postprandial	85.42 ± 4.27	74.97 ± 6.60^*^
Postpreprandial power ratio	1.03 ± 0.03	0.92 ± 0.04

^∗^ *P* < 0.05.
